# Primary Rectal Squamous Cell Carcinoma: A Rare Gastrointestinal Malignancy Presenting As Hematochezia

**DOI:** 10.7759/cureus.109195

**Published:** 2026-05-19

**Authors:** Balakrishna Ravella, Eswar Chand Gadde, Sravani Lokineni

**Affiliations:** 1 Internal Medicine, Order of Saint Francis (OSF) HealthCare St. Mary Medical Center, Galesburg, USA; 2 Internal Medicine, Ascension St. John Hospital, Detroit, USA; 3 Rheumatology, Deaconess Hospital, Evansville, USA

**Keywords:** chemoradiation therapy, definitive chemoradiotherapy, endoscopic ultrasound, hematochezia, primary rectal squamous cell carcinoma, rare colorectal malignancy, rectal mass, rectal scc

## Abstract

Primary rectal squamous cell carcinoma (SCC) is an extremely rare malignancy. We describe a 67-year-old male with a history of paroxysmal atrial fibrillation who presented with abdominal cramping, lightheadedness, fatigue, and new-onset hematochezia. Colonoscopy revealed a submucosal, ulcerated, partially obstructing mass in the distal rectum. Endoscopic ultrasound demonstrated a hypoechoic mass arising from the muscularis propria with associated lymphadenopathy on MRI of the pelvis. Biopsies confirmed moderately differentiated invasive SCC. The patient was treated with chemoradiation using 5-fluorouracil, mitomycin C, and radiation therapy. This case highlights the importance of considering rectal SCC in the differential diagnosis of rectal masses and hematochezia, as its management has shifted toward definitive chemoradiation with improved outcomes compared to traditional surgical approaches.

## Introduction

Most primary rectal cancers are adenocarcinomas. Rectal squamous cell carcinoma (SCC) is an exceedingly rare entity, with an incidence of approximately 0.1-0.3% of all rectal malignancies [1-3]. Unlike anal SCC, which arises from the squamous epithelium of the anal canal and is more strongly associated with HPV infection, primary rectal SCC originates proximal to the dentate line from glandular rectal mucosa and has less well-defined biologic drivers. The majority of colorectal SCCs (approximately 90%) arise from the anal canal rather than the rectal mucosa proximal to the dentate line [4]. True primary rectal SCC must meet strict diagnostic criteria, including absence of SCC at other sites, no extension from an anal primary, and histologic confirmation of pure SCC without glandular elements [5].

The pathogenesis of primary rectal SCC remains unclear; however, it is thought to involve squamous metaplasia of the rectal mucosa secondary to chronic irritation or inflammation, with possible contributions from human papillomavirus (HPV) infection or pluripotent stem cells capable of squamous differentiation [2,6]. Although HPV is a well-established driver of anal SCC, its role in rectal SCC is less consistent and remains incompletely understood. Patients typically present with symptoms indistinguishable from rectal adenocarcinoma, such as hematochezia, abdominal pain or cramping, changes in bowel habits, and weight loss. Despite similar clinical presentations, rectal SCC is often diagnosed at a locally advanced stage and historically has carried a poorer prognosis than adenocarcinoma [1,7].

Historically, surgical resection, such as abdominoperineal resection or low anterior resection, was the mainstay of treatment. However, recent evidence supports a paradigm shift toward definitive chemoradiation therapy (CRT) modeled after the Nigro regimen used for anal SCC, with improved survival and organ preservation rates. This therapeutic adaptation is based on histopathologic similarities between rectal and anal SCC as well as emerging retrospective outcome data demonstrating favorable disease control without immediate surgery. A systematic review by Guerra et al. (2016) demonstrated a pooled overall survival of 86% with primary CRT compared with 48% with initial surgery [1]. However, these findings are largely based on retrospective analyses and rare-disease series rather than randomized prospective trials. This case report describes a patient with primary rectal SCC diagnosed via endoscopy and endoscopic ultrasound (EUS) who was managed with CRT, underscoring the rarity of this entity and the evolving therapeutic approach.

This article was previously presented as a poster at the American College of Gastroenterology (ACG) 2021 Annual Scientific Meeting held on October 24, 2021.

## Case presentation

A 67-year-old male with a past medical history of paroxysmal atrial fibrillation not on anticoagulation and primary hypertension presented to the emergency department (ED) with a four-day history of progressively worsening intermittent bilateral lower abdominal cramping, followed by progressive fatigue and lightheadedness, culminating in two days of intermittent bright red rectal bleeding. He denied any fever, nausea, vomiting, unintentional weight loss, or prior episodes of gastrointestinal bleeding. There was no personal or family history of inflammatory bowel disease, colorectal polyps, or colorectal cancer. He was a non-smoker and denied significant alcohol use. His Eastern Cooperative Oncology Group performance status was 1.

In the ED, his initial vital signs were the following: temperature 98.7°F, heart rate 100 beats per minute, respiratory rate 18 breaths per minute, and blood pressure 118/70 mm Hg. Physical examination revealed pallor and a firm, palpable rectal mass with non-thrombosed external hemorrhoids on digital rectal examination. Laboratory evaluation demonstrated a hemoglobin of 6.5 g/dL, a drop of 6 g/dL from a baseline of 12.5 g/dL, with otherwise normal white blood cell count, differential count, platelet count, coagulation studies, basic metabolic panel, and liver function tests. The serum carcinoembryonic antigen level was within normal limits (Table 1).

**Table 1:** Laboratory values Laboratory values one week prior to admission and on the day of admission.

Laboratory parameter	One week prior	On admission	Reference range
Hemoglobin (g/dL)	12.5	6.5	13.0-17.0
White blood cell count (×10³/μL)	4.2	4.8	4.0-11.0
Platelet count (×10³/μL)	430	390	150-450
Sodium (mmol/L)	136	135	135-145
Potassium (mmol/L)	4.2	4	3.5-5.0
Creatinine (mg/dL)	0.9	1.1	0.7-1.3
Aspartate aminotransferase (U/L)	32	36	10-40
Alanine aminotransferase (U/L)	28	30	7-56
Alkaline phosphatase (U/L)	110	130	44-147
Total bilirubin (mg/dL)	1	1	0.3-1.2
Carcinoembryonic antigen (ng/mL)	-	<3.0	<3.0

**Figure 1:**
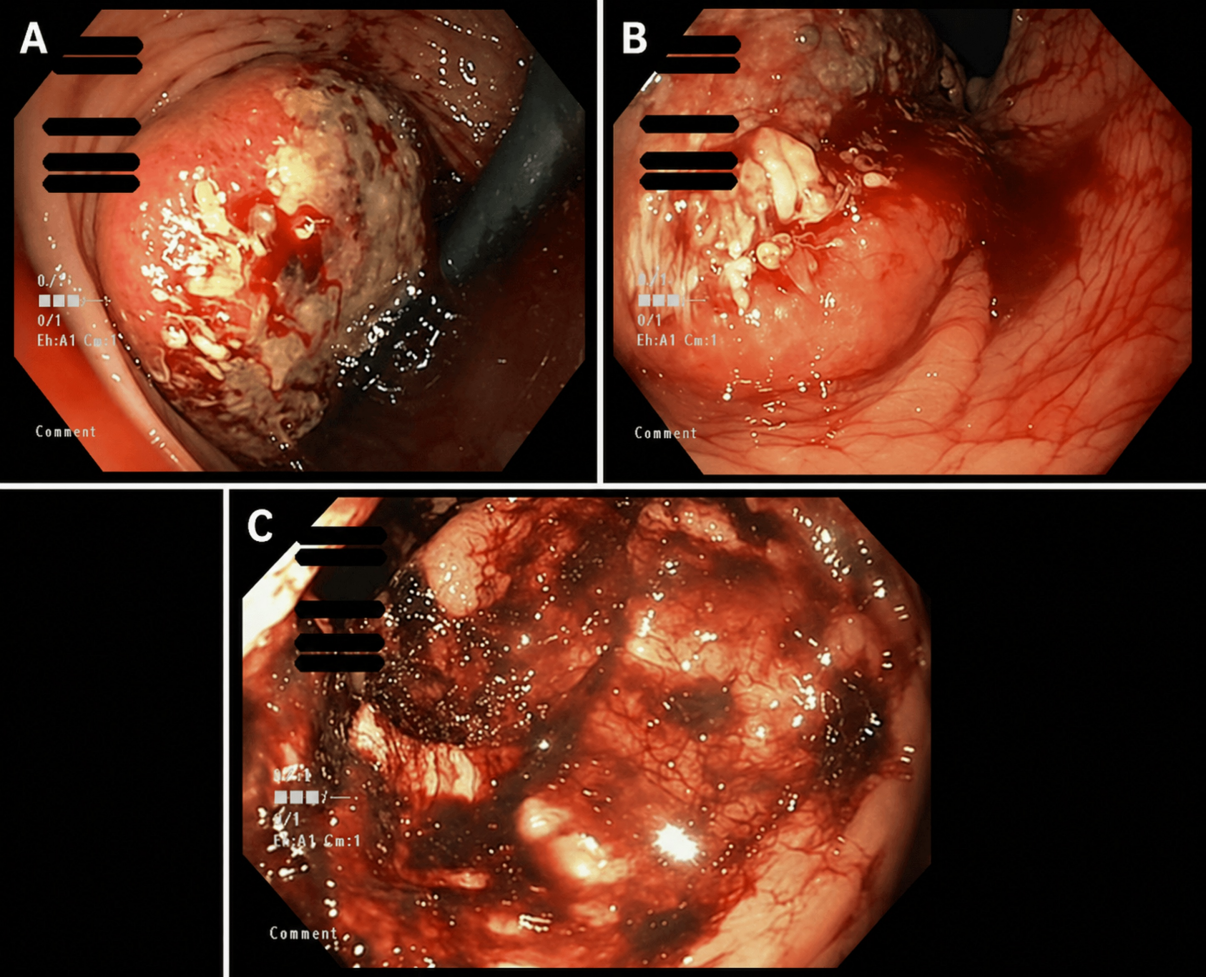
Colonoscopic views of the distal rectal mass (A) Forward endoscopic view demonstrating a large submucosal, ulcerated mass with white fibrinous exudate and areas of active oozing. (B) Retroflexed view showing the mass protruding into the lumen and its relationship to the rectal wall. (C) Additional view highlighting blood and clot within the rectal lumen secondary to active oozing from the lesion.

The patient received one unit of packed red blood cells, and a colonoscopy performed on hospital day 2 revealed a submucosal, ulcerated, partially obstructing mass in the distal rectum, approximately 1 cm proximal to the anal verge, involving roughly one-third of the luminal circumference, with active oozing (Figure 1). Its submucosal appearance initially raised concern for alternative diagnoses, including gastrointestinal stromal tumor or other mesenchymal lesions, illustrating an important diagnostic pitfall.

**Figure 2:**
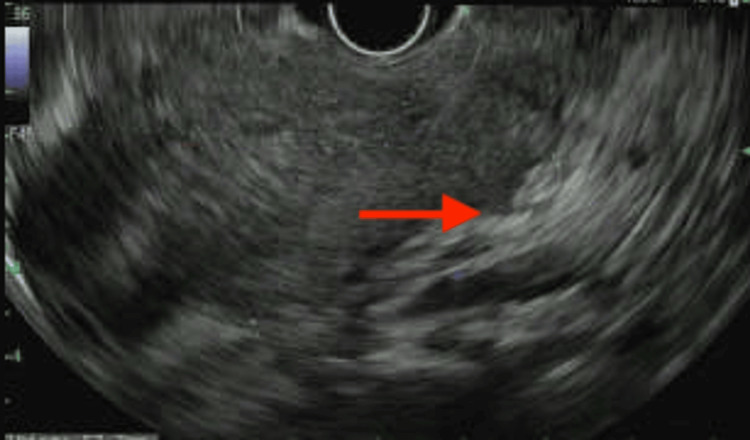
EUS image of the distal rectal mass A large, well-defined hypoechoic lesion measuring 8.5 × 5.1 × 4.0 cm is seen arising from the muscularis propria (red arrow). The hypoechoic mass disrupts the normal layered architecture of the rectal wall. EUS: endoscopic ultrasound

**Figure 3:**
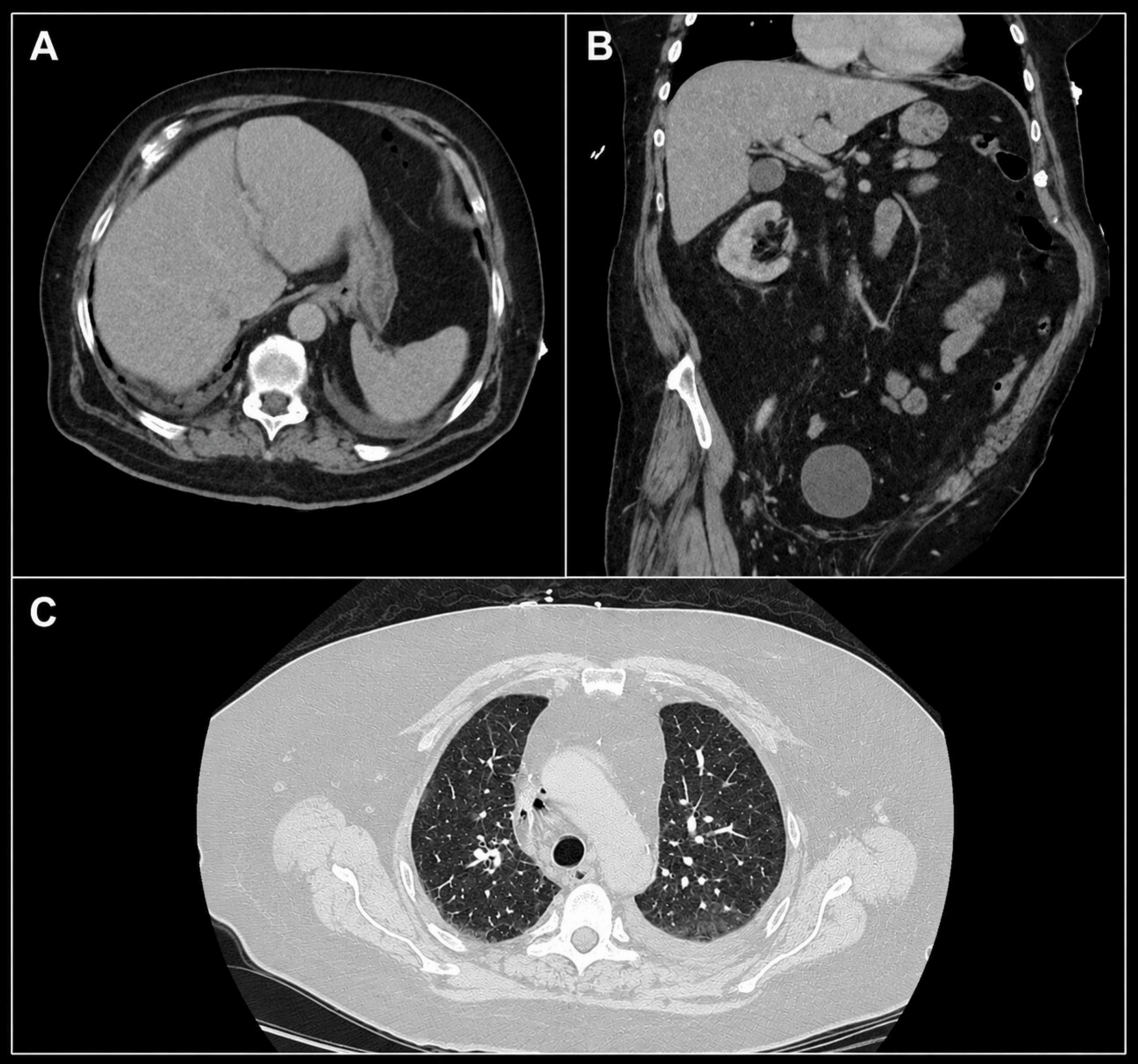
CT scan of the chest, abdomen, and pelvis (A) Axial CT image of the abdomen demonstrating no evidence of hepatic or intra-abdominal metastatic lesions. (B) Coronal CT image of the abdomen and pelvis demonstrating absence of distant abdominal or pelvic metastases. (C) CT image of the chest demonstrating no pulmonary metastases or suspicious large pulmonary nodules. CT: computed tomography

EUS performed on hospital day 2 demonstrated an 8.5 × 5.1 × 4.0 cm hypoechoic mass arising from the rectal muscularis propria (Figure 2). Pelvic MRI showed extensive mesorectal and left internal iliac chain lymphadenopathy. CT of the chest, abdomen, and pelvis revealed no evidence of distant metastasis (Figure 3). Clinical nodal staging (N1) was primarily based on pelvic MRI findings of suspicious regional mesorectal and internal iliac lymph nodes, integrated with EUS-defined local invasion, resulting in multidisciplinary staging per the American Joint Committee on Cancer 8th edition as cT3N1M0 (stage IIIA).

Endoscopic mucosal biopsies and EUS-guided fine-needle aspiration biopsies both revealed moderately differentiated invasive SCC. Immunohistochemical staining was positive for p63, CK5/6, and p40, and negative for CK20 and CDX2, confirming pure squamous differentiation without glandular components. A thorough clinical, endoscopic, and radiographic evaluation excluded metastatic SCC from a distant site or extension from an anal primary tumor. Specifically, tumor localization proximal to the anal verge, absence of anal canal mucosal involvement, and lack of distant SCC supported fulfillment of Williams' diagnostic criteria for true primary rectal SCC. HPV/p16 testing was not available in this case, which is a limitation, as the viral oncogenic contribution could not be fully assessed.

The case was discussed in a multidisciplinary gastrointestinal tumor board. The patient was initiated on definitive CRT using a regimen adapted from the Nigro protocol, consisting of continuous-infusion 5-fluorouracil, mitomycin C, and concurrent external beam radiation therapy. He tolerated the initial cycles well without major complications. At the four-week follow-up, he reported resolution of rectal bleeding and a stable hemoglobin level. While this early response is encouraging, long-term treatment response, recurrence risk, and survival outcomes remain under active evaluation with planned post-treatment restaging via endoscopy and imaging.

## Discussion

Primary rectal SCC is a rare but clinically important differential diagnosis in patients presenting with hematochezia and a rectal mass. Unlike the far more common adenocarcinoma, which comprises over 90% of rectal malignancies, rectal SCC accounts for only 0.1-0.3% of all rectal cancers and approximately 90% of colorectal SCCs [1,2]. The majority of cases arise in the distal rectum, typically within 5-10 cm of the anal verge, as observed in this patient [2,3]. True primary rectal SCC must be distinguished from anal SCC with proximal extension or metastatic disease using established diagnostic criteria proposed by Williams et al.: (1) absence of any anal canal involvement or squamous-lined fistulous tract communicating with the tumor; (2) no evidence of metastatic SCC from a distant site; and (3) histologic confirmation of pure squamous differentiation without glandular elements [4]. These criteria were satisfied in the present case through careful endoscopic localization (1 cm proximal to the anal verge), imaging, and biopsy results.

The pathogenesis of primary rectal SCC remains incompletely understood. Proposed mechanisms include squamous metaplasia of the rectal mucosa secondary to chronic irritation, inflammation, or infection, such as HPV infection, as well as malignant transformation from pluripotent stem cells within the rectal epithelium or embryonic cloacal remnants [1,2,5]. Unlike anal SCC, where HPV infection is a well-established driver in the majority of cases, the role of HPV in rectal SCC is less consistent but has been reported in select series [2,6]. Because HPV testing was unavailable in this patient, the biologic contribution of HPV could not be determined, which should be acknowledged as a diagnostic limitation. The clinical presentation is indistinguishable from rectal adenocarcinoma and includes rectal bleeding, abdominal cramping, tenesmus, changes in bowel habits, and unintentional weight loss [1,3]. Endoscopically, rectal SCC may appear ulcerated, submucosal, or polypoid and can mimic stromal lesions or lymphoma, underscoring the diagnostic utility of EUS for local staging, depth-of-invasion assessment, and tissue acquisition, as demonstrated here [2,7]. Cross-sectional imaging with pelvic MRI is essential for evaluating mesorectal and pelvic lymphadenopathy, while CT helps exclude distant metastases [1].

Historically, rectal SCC was managed similarly to rectal adenocarcinoma, with primary surgical resection (low anterior resection or abdominoperineal resection) as the cornerstone of therapy. This approach was associated with significant morbidity, including permanent colostomy in many distal tumors, and relatively poor long-term outcomes [1,8]. More contemporary evidence supports a paradigm shift toward definitive CRT, modeled after the Nigro regimen for anal SCC, using concurrent 5-fluorouracil and mitomycin C with external-beam radiation (typically 45-60 Gy) [1,2,9]. A landmark systematic review by Guerra et al., which analyzed 79 studies encompassing over 140 cases identified from the SEER database and the literature spanning 1946-2015, reported markedly superior pooled overall survival with primary CRT (86%) compared with initial surgery (48%) [1]. Local recurrence and distant metastasis rates were also significantly lower with CRT (25% vs. 10% and 30% vs. 13%, respectively) [1]. These findings have been corroborated by subsequent institutional series and reviews. Loganadane et al., in the largest prospective European case series of 23 patients with rectal SCC treated predominantly with CRT (median follow-up: 85 months), achieved an 83% clinical complete response rate, a five-year disease-free survival of 81%, a colostomy-free survival of 65%, and an overall survival of 86% [10]. Similarly, Song et al. reviewed institutional experience and literature, concluding that definitive CRT offers improved disease-related outcomes, sphincter preservation, and a more favorable morbidity profile than surgery [3]. ​​However, caution is warranted when interpreting these data, as most available evidence is derived from retrospective cohorts, pooled case series, and population-based analyses that are subject to selection bias, rather than from prospective randomized studies.

Additional population-based and retrospective data reinforce these observations. SEER analysis and systematic reviews have shown that, while rectal SCC carries a poorer prognosis than adenocarcinoma at equivalent stages (five-year overall survival is approximately 49% vs. 62%, respectively), modern CRT-based approaches have narrowed this gap through improved locoregional control and organ preservation [2]. In patients with locoregional disease (stages I-III), CRT alone or combined with surgery (when indicated for incomplete response) is now considered the preferred strategy, with surgery reserved for salvage in cases of residual or recurrent disease [1,9,10]. For metastatic (stage IV) disease, systemic therapy parallels that of anal SCC, though evidence remains limited.

This case highlights several clinically relevant points. First, the submucosal endoscopic appearance and hypoechoic EUS features arising from the muscularis propria can raise initial suspicion for stromal tumors; thus, a high index of suspicion for SCC and liberal use of EUS-guided biopsy are critical for prompt diagnosis [2,7]. Second, the patient’s locoregional presentation (mesorectal and iliac lymphadenopathy on MRI) aligns with the majority of reported cases, in which early recognition facilitates initiation of definitive CRT and avoids upfront surgery [1,10]. Finally, the ongoing treatment with 5-fluorouracil, mitomycin C, and radiation in this patient exemplifies the current standard of care, offering excellent potential for complete response and long-term disease control while preserving sphincter function.

## Conclusions

Although primary rectal SCC remains rare, clinicians should maintain a high index of suspicion in patients with rectal bleeding and rectal masses, particularly when endoscopic findings are atypical for adenocarcinoma. Prompt diagnostic evaluation with endoscopy, EUS, biopsy, and staging imaging is essential for accurate diagnosis and exclusion of anal SCC or metastatic disease. Definitive CRT has emerged as the preferred treatment strategy with favorable organ preservation potential and encouraging survival outcomes. Nevertheless, conclusions regarding superiority over surgery should remain appropriately cautious given that current evidence is predominantly retrospective. Multidisciplinary evaluation is critical, and additional prospective studies, registry analyses, and continued case reporting are needed to better define optimal management for this uncommon malignancy.
